# Particulate Matter 2.5 and Hematological Disorders From Dust to Diseases: A Systematic Review of Available Evidence

**DOI:** 10.3389/fmed.2021.692008

**Published:** 2021-07-14

**Authors:** Kamonpan Fongsodsri, Supat Chamnanchanunt, Varunee Desakorn, Vipa Thanachartwet, Duangjai Sahassananda, Ponlapat Rojnuckarin, Tsukuru Umemura

**Affiliations:** ^1^Department of Tropical Pathology, Faculty of Tropical Medicine, Mahidol University, Bangkok, Thailand; ^2^Department of Clinical Tropical Medicine, Faculty of Tropical Medicine, Mahidol University, Bangkok, Thailand; ^3^Information Technology Unit, Faculty of Tropical Medicine, Mahidol University, Bangkok, Thailand; ^4^Division of Hematology, Department of Medicine, Faculty of Medicine, King Chulalongkorn Memorial Hospital, Chulalongkorn University, Bangkok, Thailand; ^5^Department of Medical Technology and Sciences, International University of Health and Welfare, Ohkawa, Japan

**Keywords:** air pollution, particulate matter, anemia, leukemia, thrombosis, blood coagulation

## Abstract

Particulate matter 2.5 (PM_2.5_) in the air enters the human body by diffusion into the blood. Therefore, hematological abnormalities might occur because of these toxic particles, but few studies on this issue have been reported. According to Cochrane guidance, we performed a systematic review on the relationship between exposure to PM_2.5_ and the risk of hematological disorders. Ten articles were included in this review. Anemia was found among children and elderly populations with 2- to 5-year PM_2.5_ exposure. Young children from mothers exposed to air pollution during pregnancy had a higher incidence of leukemia similar to the elderly. Supporting these data, outdoor workers also showed abnormal epigenetic modifications after exposure to very high PM_2.5_ levels. Adults living in high PM_2.5_ areas for 2 years were more likely to develop thrombocytosis. Finally, elderly populations with 7- to 8-year PM_2.5_ exposure showed increased risks of venous thromboembolism. In conclusion, the associations between PM_2.5_ and hematological aberrations among high-risk people with long-term exposure were reported.

## Introduction

Air pollution is one of the most important environmental and health problems worldwide (1, 2). Thailand and other countries have been encountering excessive amounts of particulate matter (PM) primarily from combustion of fuels, coal, and natural gases. PMs are complex mixtures of airborne particles with differences in chemical compositions, sizes, and origin (3). They can be classified into coarse (PM_10_; diameter range 2.5–10 μm), fine (PM_2.5_; size <2.5 μm), and ultrafine particulate (UFP; size <100 nm) matter (4–6). Both PM_10_ and PM_2.5_ concentrations are widely measured in the environment. After human exposure to PM, PM_10_ remains in the nasal cavities and upper respiratory tracts, but PM_2.5_ can reach the alveoli and then penetrate into the blood (3, 7–10). Fine PM can directly cause health problems such as respiratory (reactive airway, lung cancer, and chronic obstructive pulmonary diseases), cardiovascular (heart failure and myocardial infarction), and allergic diseases (rhinitis and eczema) (9, 11). In 2013, the International Agency for Research on Cancer (IARC) of the World Health Organization (WHO) reported that outdoor air pollution is toxic to humans (12). The annual mean PM_2.5_ level over 10 μg/m^3^ or 24-h mean over 25 μg/m^3^ was defined as deleterious to the health of the population (13). Children and women are high-risk groups that are prone to develop cardiovascular diseases (18–27%) and pulmonary diseases (8–20%) from PM exposure (14). As PM_2.5_ can be absorbed from the respiratory tract into the blood, they may interact with blood cells and plasma components (7, 10). PM_2.5_ also affects red blood cells, white blood cells, or platelet functions, and therefore, some patients may acquire hematological diseases. Apart from exposure to PM_2.5_, other risk factors also play synergistic roles in causing the diseases. Therefore, high-risk groups for developing hematologic diseases need to be identified. In this review, we aim to study the relationship between PM_2.5_ exposure and risks for abnormal hematological parameters.

## Methods

**Figure 1:**
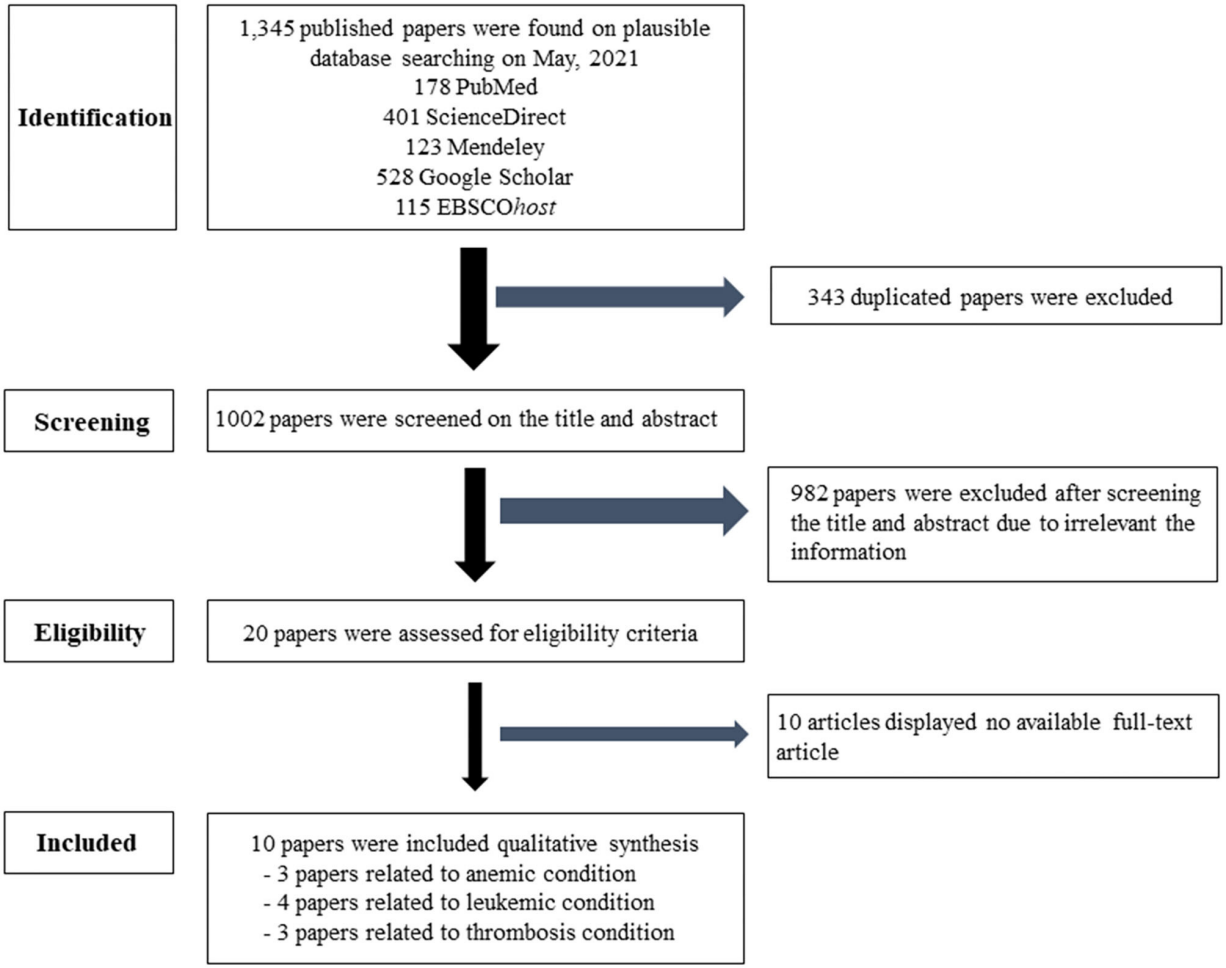
Flow chart of the selection criteria. Ten eligible articles were included and extracted data for this article.

This systematic review followed the Cochrane guidelines. The search used PubMed®, Science Direct, Mendeley, Google Scholar, and EBSCO*host* database. The keywords were separated into three categories. Searching group1 used “air pollution” or “particulate matter” or “PM_2.5_” and “anemia.” In group2, the terms “air pollution” or “particulate matter” or “PM_2.5_” and “leukemia” were searched. In group3, “air pollution” or “particulate matter” or “PM_2.5_” and “thrombosis” or “coagulation” were used. In group4, “air pollution” or “particulate matter” or “PM_2.5_” and “lymph nodes” were used. In group5, “air pollution” or “particulate matter” or “PM_2.5_” and “spleen” were added. The inclusion criteria were studies on PM_2.5_ in relation to hematological diseases, such as anemia, leukemia, and thrombosis in humans, and publications between 2014 and 2021. Duplicated articles, irrelevant titles, the non-English languages, and unavailable full papers were excluded. Afterwards, eligible articles were archived to the EndNote X7.7.1 for Windows (Thomson Reuters, USA). Interesting information from matched articles of the PM_2.5_ related to hematologic diseases were extracted to identify the risk factors and assessed by three independent hematologists. The search algorithms are shown in Figure 1.

## Results

### Study Characteristics

Table 1 shows the characteristics of the 10 available studies included in this systematic review. Of these, there were three studies using the panel design, four case–control studies, two cohort studies, and one cross-sectional study. Sample sizes of the population per study ranged from 4,121 to 139,368 for the panel design and 453.413 for the cross-sectional study. Most studies included subjects over 35 years of age. The studies were located in Peru, Denmark, the United States, Italy, Canada, China, and Taiwan. Air pollutant data were obtained from fixed sites or personal systems or both. Three studies reported the results related to erythrocyte disorders, four studies related to white blood cells, and three studies demonstrated results associated with platelets or thrombotic conditions.

**Table 1:** Characteristics of included studies.

**References**	**Study design**	**Location**	**Period of study**	**Population**	**Sample size**	**Age, year (%)**	**Exposure measurement**	**Results**
Taj et al. (15)	Case–control	Denmark	1979–2014	Adult	66,596	≥20	Ambient	Leukemia cases
Hvidtfeldt et al. (16)	Case–control	Denmark	1981–2013	Children	779	0–19	Ambient	Non-Hodgkin lymphoma
Morales-Ancajima et al. (17)	Panel	Peru	2012–2016	Children	139,368	6–59 mo	Ambient	Hemoglobin level
Zhang et al. (2)	Cohort	Taiwan	1996–2014	Adult	794,125	41	Ambient	Platelet level
Honda et al. (18)	Panel	America	Wave 1, 2005–2006 Wave 2, 2010–2011	Elderly	4,121	69.6	Ambient	Hemoglobin level
Lavigne et al. (19)	Retrospective cohort	Canada	1988–2012	Pregnant and toddlers	2,350,898	29.4 and age <1	Ambient	White blood cell levels
Visani et al. (3)	Case–control	Italy	2013–2015	Adult	38	65 (range 20–71)	Ambient	Particulate toxin levels
Sanchez-Guerra et al. (20)	Case–control	China	2008	Adult	120	39–46 (25.9)	Ambient and personal	5hmC and 5mC levels
Kloog et al. (21)	Cross-sectional	America	2000–2008	Elderly	453,413	79	Ambient	Thrombosis event
Brook et al. (22)	Panel	Canada	2000–2010	Elderly	N/A	>75	Ambient	Thrombosis event

### Hematologic Disorders (Anemia, Leukemia, and Thrombosis)

#### Is PM_2.5_ a Risk Factor for Anemia?

A few studies linked PM_2.5_ exposure to erythrocyte disorders, especially anemia. According to the WHO criteria, anemia was defined as a hemoglobin (Hb) level lower than 13.0 g/dl in men, 12.0 g/dl in women, and 11.0 g/dl in children (23, 24). A study by Morales-Ancajima et al. (17) was performed in 139,368 children (age 6–35 months) to evaluate the association between Hb and air pollution in their residential areas. Hb levels decreased among children who were exposed to PM_2.5_ (range 24.97–28.84 μg/m^3^) during the 4-year study. Mild to moderate anemia in children was related to areas with high 24-h PM_2.5_ concentrations with the means between 25 (25) and 50 μg/m^3^ (26). Moderate to severe anemia in children were associated with very high 24-h mean PM_2.5_ of over 50 μg/m^3^ [odds ratio (OR) = 2.83, 95% confidence interval (CI) 1.39–5.75] (27). Anemic children were detectable in 30.8% of the population, while moderate to severe anemia was observed in 8.8% of the population (17). Similarly, Honda et al. (18) showed that 4,121 elderly people with anemia (34.9%) lived in areas with high annual mean PM_2.5_ levels (>11.1 ± 2.8 μg/m^3^). Interestingly, elderly subjects with 2- to 5-year exposure to PM_2.5_ pollution showed declines in Hb levels of approximately 0.81 ± 0.06 g/dl. Honda et al. (18) explained the relationship between air pollution and anemia in the elderly population with elevated C-reactive protein (CRP) levels that indicated chronic inflammatory responses to PM_2.5_. In an animal model, the increased harmful effects of PM_2.5_ exposure to young rather than adolescent mice were mediated by the impairment of bone marrow microenvironment (27, 28). The mechanism of PM_2.5_ action is probably a reactive oxygen species formation which increases inflammatory cytokines (TNF-α, IL-1β, and IL-6) in the cells. Inflammation may inhibit differentiation and proliferation of erythroid precursor cells and enhance an erythropoietin (Epo) resistant state (27–29). Another potential explanation is that inflammatory cytokines can upregulate hepcidin synthesis causing breakdown of ferroportin, thus reducing iron absorption in the gastrointestinal tract (30, 31). In another mouse model, an association between particulate matters (PM_2.5_ and PM_0.1_) and the deformation of murine erythrocytes was found (6). The mice exposed to PM_2.5_ (100 s for 8 days) increased erythrocyte distortion, which finally led to hemolytic anemia (6). Anemia observed in mice exposed to air pollution showed significant dose and time dependency (27).

#### How Is PM_2.5_ Related to Leukemia?

It has been known since 1997 that gasoline pollution is one of the factors in gene–toxin–environmental interactions promoting leukemogenesis (32, 33). Leukemia is a malignant clonal disease that results in abnormal proliferation and impaired cellular differentiation of hematopoietic stem cells. Multiple myelotoxicity substances commonly injure hematopoietic cells (3). Exposure to environment toxins might be one of the etiologies of childhood leukemia (34). Acute leukemia represents approximately 30% of pediatric and 25% of adult malignancies (3, 35, 36). A study by Visani et al. (3) measured the particulate toxin in blood samples of adult patients (age range 20–71 years) with acute myeloid leukemia (AML). The levels of PM_2.5_ were higher among AML patients compared to healthy controls (3). Similarly, Taj et al. (15) demonstrated that PM_2.5_ components were more positively associated with AML [OR = 1.14; interquartile range (IQR) = 1.00–1.29] than chronic myeloid leukemia and chronic lymphocytic leukemia. The effects of PM_2.5_ and other components are related not only to adult leukemia but also to childhood hematological malignancy as reported in studies by Lavigne et al. (19) and Hvidtfeldt et al. (16). Consistently, Lavigne et al. (19) demonstrated the relationship between traffic air pollution exposure in approximately a million pregnant women and the incidence of childhood acute lymphoblastic leukemia (ALL) in their offspring. They found a high hazard ratio in exposure during the first trimester of pregnancy [hazard ratio (HR) = 1.20, 95% CI = 1.02–1.41] (19). This study design showed the strongest relationship between fetal exposure to air pollution during pregnancy and leukemia in the first year of age. A study by Hvidtfeldt et al. (16) showed a high OR ratio of 2.05 (IQR = 1.10–3.38) in childhood non-Hodgkin lymphoma with exposure to PM_2.5_. Molecular epidemiology studies suggested that DNA methylation of the leukemic gene was positively correlated with exposure to environmental toxins (37, 38). DNA methylation incorporates a methyl group to the position of the fifth carbon of cytosine to produce 5-methylcytosine (5mC). Subsequently, 5mC is oxidized into 5-hydroxymethylcytosine (5hmC) which suppresses gene expression. Both 5mC and 5hmC are markers for DNA methylation in malignancy cells (39, 40). A study by Sanchez-Guerra et al. (20) demonstrated the increase in methylated genomic contents (5hmC and 5mC) among subjects who were exposed to PM_10_ and PM_2.5_ for 4–7 work-days. Blood samples of 60 outdoor workers exposed to PM_10_ showed significantly higher 5hmC levels (*p* = 0.001 at 4 work-days, *p* = 0.005 at 7 work-days, and *p* < 0.001 at 14 work-days) and increasing 5mC levels in people exposed to PM_2.5_ (*p* = 0.005). This study is limited by the exposure times to PM_2.5_. In addition, high 5hmC and 5mC levels, which indicated epigenetic modification, were detectable among people in Beijing (20). The mechanism of PM_2.5_ in AML development could be explained by specific proteins. Protein-forming nanoparticles, termed corona proteins, that cover PM_2.5_ particles can alter epigenetics and tumor suppressor gene expression (3, 15, 41, 42). DNA methylation is highly sensitive to environmental PM_2.5_ exposures (16, 20). Therefore, this modification can inhibit gene expression, impeding cellular differentiation as one of the steps of leukemic development. Long-term exposure to PM_2.5_ might alter both gene-coding and non-coding DNA methylation (20). An *in vitro* study by Jin et al. (43) found that the progression of leukemic cells was induced by prolonged exposure to PM_2.5_. The proposed mechanism is the reactive oxygen species-mediated pathway (43). Moreover, the PM_2.5_ component had been classified as a carcinogenic class I agent since 2013 (3). Apart from PM_2.5_ particles, its components, i.e., aluminum, black carbon, sulfur, lead, titanium, and silicon, may be involved in the pathogenesis of leukemia (20). In conclusion, a positive relationship between PM_2.5_ and leukemic cells is suggested by epidemiological evidence, and the molecular mechanisms are used to explain the pathophysiology of diseases.

#### Can Thrombosis and Coagulation Be Promoted by PM_2.5_?

Evidence from related studies links particulate matters to platelet function (2, 4, 22, 44–46). The American Heart Association-AHA (2004) proposed that thrombotic mechanisms may be explained by daily exposure to PM_2.5_ among patients with atherosclerotic cardiovascular disorders (22). Thrombosis is the most common pathology in patients with acute cardiac ischemia and stroke on top of atherosclerosis (4, 47, 48). Brook et al. (22) found that deep venous thrombosis (DVT) and hypercoagulability were also the results of long-term (almost 8-year) exposure to PM_2.5_. Similarly, the Zhang et al. (2) study was performed with 175,959 men (with 396,248 observations) and 186,437 women (with 397,877 observations) correlating with 2-year average PM_2.5_ concentrations. They found a relationship between thrombocytosis (men, 0.42% and women, 0.49%) and increment in PM_2.5_ (every 10 μg/m^3^ increase of PM_2.5_) (2). In the United States, a study by Kloog et al. (21) found a slight increment in DVT (0.63%, 95% CI = 0.03–1.25) but no significant increase in pulmonary embolism (PE), after short-term exposure to PM_2.5_. Interestingly, there were increased risks of DVT (6.98%; 95% CI = 5.65–8.33) and PE (2.67%; 95% CI = 5.65–8.33) events after long-term exposure to PM_2.5_ (21). The mechanism of thrombosis by PM_2.5_ may be from increased inflammatory cytokine (IL-6) levels, oxidative stress, platelet activation, stimulated coagulation pathway, and reduced fibrinolysis (3, 46, 49, 50). These conditions are known to promote thrombotic phenomena in humans (51). In animal models, there is a study on the effects of PM_2.5_ that can cause disseminated intravascular coagulation (DIC) through coagulation activation in rats (46). Liang et al. (46) found that rat exposure to PM_2.5_ increasingly expressed inflammatory cytokines, IL-6, IL-1β, and CRP in plasma. Moreover, tissue factor-dependent extrinsic pathways coagulation systems, as well as expression of adhesion molecules, such as VCAM-1 and ICAM-1, were upregulated after moderate to high doses of PM_2.5_ (average 35 μg/m^3^ for 30 exposure days). Finally, a shift of hemostatic balance to a pro-thrombotic/pro-coagulation state is induced by exposure to PM_2.5_ (4).

## Conclusions

High PM_2.5_ exposure is one of the most important avoidable hazards to human health. The effects on hematological systems have been less well-studied. There were fewer than 20 available entries in the web search according to the Cochrane recommendation as the damages are not easily analyzed and are mostly unnoticed. All available studies found harmful effects of long-term exposure to PM_2.5_ to the hematopoietic system. Hematological parameter changes in people exposed to medium to high PM_2.5_ concentrations for more than a year are summarized in Table 2. The pathophysiology of PM_2.5_ pollution may be from increased inflammatory responses affecting each hematological component. For the public, avoidance of exposure and reduction in PM_2.5_ pollution should be encouraged. Interventions should be focused on the high-risk groups including the elderly with cardiovascular diseases, young children, and pregnant women. Personal facemasks can minimize inhaled small particles and reduce pollution exposure time. Air purifiers also lessen indoor pollution concentrations. Improving air quality has to be addressed for the general population. Decreasing PM_2.5_ pollution is essential to prevent hematological adverse events. Further studies are needed to determine whether reducing PM_2.5_ exposure can decrease these disorders.

**Table 2:** Pathophysiology of PM_2.5_ effects on individual hematologic parameters and the high-risk groups.

**Parameters**	**Pathophysiology of actions**	**Effects**	**High-risk groups (reference)**
Red blood cells (RBCs)	Increased inflammation - Ineffective erythropoiesis - Downregulation of erythropoietin - Reduction of iron absorption and recycling	Anemia	- Children (17) (Mild/moderate anemia; 4-year exposure to high PM_2.5_ levels^a^) (Moderate/severe anemia; 4-year exposure to very high levels^b^) - Elderly (18) (2- to 5-year exposure to high PM_2.5_ levels^a^)
White blood cells (WBCs)	Leukemogenesis - Inflammation with epigenetic modifications - Reactive oxygen species	Leukemia and lymphoma	- Young children exposure *in utero* (16, 19) (1-year exposure of unidentified actual PM_2.5_ levels) - Outdoor workers (20) (PM_2.5, 10_ levels for 4 to 7 work-days) - Adult age above 60 years (15) (10-year exposure to PM_2.5_)
Platelets and coagulation	Inflammation causing platelets activation, stimulated coagulation pathway, oxidative stress, reduced fibrinolysis, and vascular endothelial injury	Thrombocytosis and thrombosis	- Adult with thrombocytosis (2) (2-year exposure to high PM_2.5_ levels^a^) - Elderly with deep vein thrombosis (21, 22) (7- to 8-year exposure to high PM_2.5_ levels^a^) - Adults with pulmonary embolism (21) (7-year exposure to high PM_2.5_ levels^a^)

a *High PM_2.5_: A 24-h mean level between 25 and 50 μg/m^3^ or annual mean over 10 μg/m^3^*.

b *Very high PM_2.5_: A 24-h mean level over 50 μg/m^3^*.

## Data Availability Statement

The original contributions presented in the study are included in the article/supplementary material, further inquiries can be directed to the corresponding author/s.

## Author Contributions

KF and SC: conception of the article and updating of the article as per the suggestions. KF, SC, VD, VT, and DS: writing-first draft preparation. KF, SC, VD, VT, DS, PR, and TU: writing-review and editing. All authors have read and agreed to the published version of the manuscript.

## Conflict of Interest

The authors declare that the research was conducted in the absence of any commercial or financial relationships that could be construed as a potential conflict of interest.
